# Acute Cerebral Ischemia Temporally Associated with Marijuana Use

**DOI:** 10.7759/cureus.5239

**Published:** 2019-07-25

**Authors:** Vyas Viswanathan, Crystal Yu, Jacob A Sambursky, Supreet Kaur, Alexis N Simpkins

**Affiliations:** 1 Neurology, University of Florida, Gainesville, USA; 2 Neurology, Albany Medical Center, Albany, USA; 3 Neurology, University of Miami Miller School of Medicine, Miami, USA

**Keywords:** marijuana, cbd, thc, cannabis, stroke, ischemia, vasculopathy, cerebrovascular

## Abstract

The cerebrovascular effects of marijuana use are not well described. With increasing legalization of cannabis for medical and recreational use in North America, identification of potential risks of the drug is necessary. We present the case of a 31-year-old man who had two ischemic infarctions in different vascular distributions, without other identifiable etiology, which were temporally associated with marijuana use. We additionally identified the level of metabolites in his system and discussed the need for a systematic description of these cases to determine whether a dose-dependent effect exists.

## Introduction

The incidence of stroke in young adults has increased worldwide over the past two to three decades, with strokes of undetermined etiology accounting for 20% to 30% of cases [1]. The burden of stroke in this population is significant, given the long life expectancy of these patients after stroke, the resulting disability in their most productive years, and the need for long-term care. Increasing incidence may be in part due to improved stroke detection, greater prevalence of known stroke risk factors, and more recreational and illicit drug use [1].

Marijuana use has greatly increased in the last few years in the United States. The National Institute on Alcohol Abuse and Alcoholism reports the prevalence of marijuana use in 9.5% in 2013, double the decade prior [2]. This may be partly due to the increasing legalization of medical marijuana, decriminalization of recreational marijuana, and the decreased perception of risk regarding marijuana use [3]. As decriminalization and legalization efforts continue, increased prevalence of marijuana use can be expected. Despite the increasing availability, its long-term effects on health are poorly understood. It is unclear whether the act of smoking marijuana, active compounds within marijuana, or other substances mixed with marijuana can increase stroke risk. It will be important to identify if marijuana exposure increases the risk of cerebrovascular disease.

We describe a young man who had multiple ischemic infarctions separated by time and vascular territories without any other causes of stroke identified. We hypothesize that he may have increased his stroke risk due to his use of inhaled marijuana.

## Case presentation

A 31-year-old Caucasian male presented to the emergency room one hour after the sudden onset of left hemiparesis, left facial droop, and slurred speech a few minutes after smoking marijuana. His past medical history included daily cannabis use, daily nicotine inhalation via electronic (e-) cigarettes, hypertension, and a right superior cerebellar artery stroke nine months prior to admission without residual deficits. His prior ischemic stroke also reportedly occurred within a few minutes of smoking marijuana. Work-up for the prior stroke was performed at an outside hospital and was unrevealing for a cause per history.

**Figure 1:**
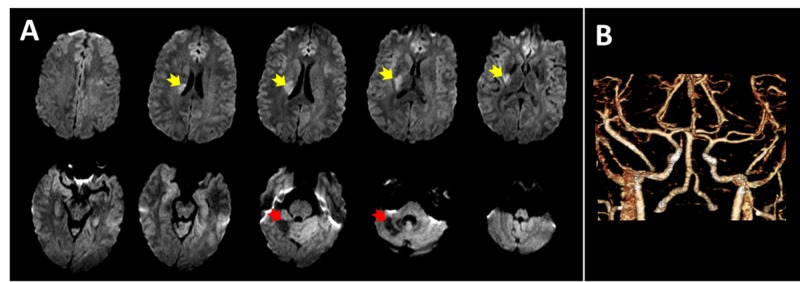
Imaging of the brain and vasculature A) Diffusion-weighted imaging of the brain demonstrates acute ischemia in the right periventricular white matter, internal capsule, and basal ganglia (yellow arrows). The patient’s prior stroke in the right cerebellum was also seen (red arrows); B) No focal stenosis, occlusions, or other vasculopathy was identified on computed tomography angiography.

On exam, the patient was afebrile with vital signs within normal limits. He had fluctuating symptoms ranging from mild drift in the left upper and lower extremities to complete paralysis of the same extremities, left facial droop, and dysarthria. Computed tomography (CT) of the head did not demonstrate any acute bleeds. The patient received tissue plasminogen activator and was admitted to the neuro-intensive care unit. Magnetic resonance imaging of the brain confirmed an ischemic stroke of the right periventricular white matter extending to the posterior limb of the internal capsule and posterior right basal ganglia and additionally showed encephalomalacia in the right superior cerebellum from his prior stroke (Figure 1A). Vessel imaging did not show any intracranial or extracranial vascular abnormalities or perfusion defects (Figure 1B).

The patient’s urine drug screen was only positive for cannabinoids, and his urine 11-nor-delta-9-THC-carboxylic level was very high at 984 ng/ml. Stroke labs including the lipid profile, syphilis screen, and hemoglobin A1c level were within normal limits. The patient had elevated erythrocyte sedimentation rate (61) and C-reactive protein (21.5), but a vasculitis and hypercoagulable workup including HIV testing, antinuclear antibody, antineutrophil cytoplasmic antibody, antiphospholipid antibody, Dilute Russel’s Viper Venom Time, anti-Ro antibody, and anti-La antibody were negative.

Trans-thoracic echocardiogram with bubble study and transesophageal echocardiogram were normal. Cardiac monitoring showed normal sinus rhythm without arrhythmias. Outpatient cardiac monitoring after his first stroke did not reveal any abnormal heart rhythms, and the patient refused repeat testing. CT of the chest, abdomen, and pelvis and testicular ultrasound were negative for occult mass lesions.

At discharge, the patient was asymptomatic and back to baseline. On six-month follow-up, he remained symptom-free. He has stopped smoking marijuana but continues to use e-cigarettes.

## Discussion

Marijuana is being decriminalized and legalized in many regions of the United States. Despite the increasing availability of marijuana, its effects on cerebrovascular health are unclear. Although the mechanistic relationship between the chemical components of marijuana and stroke continues to be investigated, clinical cases of stroke associated with marijuana use have been published for over 30 years. Reports often cite a temporal relationship between the onset of stroke symptoms and ingestion of marijuana without other identifiable stroke risk factors in young adults, though cardiac arrhythmia or tobacco use is often present [4-7].

The incidence of acute ischemic stroke in marijuana users aged 15-54 years was 0.4% in the Nationwide Inpatient Sample database from 2004 to 2011 [8]. While the overall incidence is low, there may be a subset of patients at elevated risk, in part due to epigenetic factors, variable effects of delta-9-tetrahydrocannabinol (THC) and cannabidiol (CBD), non-systematic toxicology screens, and possible dose dependency [9-12].

We present the case of a young man, who developed acute ischemic strokes in distinct vascular territories on multiple occasions soon after smoking marijuana. The case is unique because after an extensive evaluation for causes, including screening for occult tumors, the patient’s primary risk factor was long-term heavy cannabis use.

Literature is inconsistent in the role of marijuana in acute ischemia. Two prospective trials evaluating the effect of marijuana exposure on stroke did not show an association between marijuana and stroke, though were found to have moderate to high risk of bias due to minimal exposure and long follow up time without re-assessment of exposure [13,14]. A case-controlled study found 2.3 times increased odds of having a stroke compared to non-users, though the effect was not present when controlling for tobacco use [4]. On the other hand, a national database study found 1.17 times increased risk of stroke with cannabis exposure even when controlling for tobacco [8]. Several case reports document a temporal association of marijuana and strokes in young adults with otherwise cryptogenic strokes [5-6,12]. Several of these cases did not have confounding exposure to alcohol, cigarettes, or other vasoactive compounds [12].

Whether the relationship between marijuana exposure and stroke is dose dependent is unclear. In a non-systematic review of literature on marijuana exposure and strokes, 86% of all strokes were in chronic cannabis users [12]. Daily heavy cannabis users had similar urine concentrations of marijuana metabolite as our patient [15]. To our knowledge, the concentration of marijuana in blood or urine has only been reported in stroke literature once in the case of a 27-year-old with extremely high serum metabolite levels who developed a basal ganglia intracerebral hemorrhage [6]. Interestingly, in both our case and the one previously reported, vasculopathy was not observed on vessel angiography, and the patients did not have hemodynamic instability on presentation.

In heavy cannabis users, strokes may occur due to similar mechanisms as smoking cigarettes. Smoking cigarettes and second-hand smoke exposure are well-known risk factors for stroke. The possible mechanisms include increased platelet aggregation, atherogenesis, and toxic compounds accelerating atherosclerosis [16]. Emerging evidence suggests that smoking marijuana may contribute to similar mechanisms of increasing stroke risk by causing oxidative stress, endothelial cell dysfunction, and inflammation and coagulation disturbances [17]. Whether the act of smoking marijuana is a greater stroke risk than other forms of use is unknown, though out of 85 patients who had strokes after marijuana exposure, only one individual ingested the drug [7,12]. The patient had significant hemodynamic instability, cerebral vasospasm, and atrial fibrillation following ingestion of cannabis tablets with alcohol [7].

Hypotension, cardioembolism from a tachyarrhythmia, reversible cerebral vasoconstriction syndrome, and vasculopathy are possible mechanisms for strokes in patients with recent cannabis use [12]. The two most abundant active compounds in marijuana are THC and CBD, both of which are known to have cardiovascular effects in opposition to one another. THC can cause tachycardia and hypertension while CBD can cause bradycardia and hypotension [10]. Research in animal models suggests beneficial effects on the cardiovascular system such as maintaining lower heart rate and blood pressure after global hypoxia and increasing blood flow during reperfusion after ischemia [11]. In humans, smoking marijuana increased heart rate in a dose-dependent fashion despite the subjective perception of intoxication not being dose-dependent [18]. In the only reported case of marijuana ingestion resulting in stroke, the patient had significant hemodynamic instability and atrial fibrillation, though it is unclear if this may place chronic users at higher risk of developing transient cardiac arrhythmias resulting in cardioembolic strokes.

Of note, the patient in the present report smoked e-cigarettes containing nicotine, which has an unclear role in cardiovascular disease. Nicotine replacement therapy, albeit vasoactive, is not associated with an increased risk of MI, stroke, or death [19]. Furthermore, the health effects of e-cigarettes and vaporized nicotine consumption are not well studied. A 2014 policy statement from the American Heart Association remains equivocal on the general health effects of e-cigarettes due to the recent increase in use and limited research on long-term outcomes. However, short-term use in healthy individuals had no adverse effects [20].

Multiple states have legalized marijuana for medical and recreational use. As the number of states approving legalization increases, the perceived harm of marijuana use by the general public will likely decrease with increasing accessibility. There are still many important issues to address regarding the poorly understood association between marijuana and its long-term health risks.

## Conclusions

Despite public perception that marijuana is safe for use, there is a need for equipoise based on clinical evidence. A systematic approach to describing and investigating marijuana-related cerebrovascular pathology is necessary. Future research should focus on toxicology screening and reporting of serum or urine concentrations of marijuana metabolites, investigation of dose-related response, and evaluation of the effect of both THC and CBD on human hemodynamics, and examining the role of different modes of drug delivery.
